# Characterization of the complete chloroplast genome of *Epimedium brevicornu* (Berberidaceae)

**DOI:** 10.1080/23802359.2019.1678429

**Published:** 2019-10-22

**Authors:** Guoqi Zheng, Chen Zhang, Juan Yang, Xing Xu

**Affiliations:** a School of Life Science, Ningxia University, Yinchuan, China;; b Breeding Base for State Key Laboratory of Land Degradation and Ecological Restoration in Northwest China, Ningxia University, Yinchuan, China

**Keywords:** *Epimedium brevicornu*, genus Epimedium, chloroplast genome, phylogenetic tree

## Abstract

The complete chloroplast genome sequence of *Epimedium brevicornu,* a common medicinal plant is widely distributed in South China. The plastome is 156,947 bp in length, with one large single-copy region of 88,535 bp, one small single-copy region of 17,012 bp, and two inverted repeat (IR) regions of 27,700 bp. It contains 132 genes, including 83 protein-coding genes, 8 ribosomal RNA, and 38 transfer RNA. Phylogenetic tree shows that this species is a sister to *Epimedium acuminatum*. The published plastome of *E. brevicornu* provides important insight into conservation and restoration efforts for *E. brevicornu*.

*Epimedium brevicornu* Maxim. belongs to the genus *Epimedium* of the Berberidaceae. Members of this genus are widely distributed in China, are well known as exotic perennial garden plants in western countries and important medicinal herbs in Asian countries (Stern 2002). In the last decades, the use of *E. brevicornu* in traditional Chinese medicine has led to a rapid increase in the information available on the active components of *E. brevicornu* (Li 1991; Wang et al. 2004; Meng et al. 2005). However, due to anthropogenic over-exploitation and decreasing distributions, this species needs urgent conservation. Knowledge of the genetic information of this species would contribute to the formulation of protection strategy. In this study, we assembled and characterized the complete chloroplast (cp) genome sequence of *E. brevicornu* based on high-quality pair-end sequencing data.

Fresh leaves of *E. brevicornu* were collected from Liupanshan mountain (Guyuan, Ningxia, China; coordinates: 106°25′E, 35°33′N). Dried and kept in silica gel for DNA extraction and then stored in the Herbarium of the College of Life Science, Ningxia University with the accession number of ND190613025.Total genomic DNA was extracted with a modified CTAB method (Doyle and Doyle 1987). First, we obtained 10 million high-quality pair-end reads for *E. brevicornu*, and after removing the adapters, the remained reads were used to assemble the complete chloroplast genome by NOVOPlasty (Dierckxsens et al. 2017). The complete chloroplast genome sequence of *E. pseudowushanense* was used as a reference. Plann v1.1 (Huang and Cronk 2015) and Geneious v11.0.3 (Kearse et al. 2012) were used to annotate the chloroplast genome and correct the annotation. The complete cp genome sequence was deposited in GenBank under accession number MN381716.

The *E. brevicornu* cp genome is 156,947 bp in length, exhibits a typical quadripartite structural organisation, consisting of a large single copy (LSC) region of 88,535 bp, two inverted repeats (IR) regions of 25700 bp and a small single copy (SSC) region of 17,012 bp. The cp genome contains 132 complete genes, including 83 protein-coding genes (83 PCGs), 8 ribosomal RNA genes (4 rRNAs), and 38 tRNA genes (38 tRNAs). Most genes occur in a single copy, while 15 genes occur in double, including all rRNAs (4.5S, 5S, 16S, and 23S rRNA), 7 tRNAs (*trnA-UGC, trnI-CAU, trnI-GAU, trnL-CAA, trnN-GUU, trnR-ACG*, and *trnV-GAC*), and 4 PCGs (rps7, ndhB, ycf2 and rpl23), while one partial *ycf1*, *infA,* and *rpl2* genes were identified as a pseudogene. The overall AT content of cp DNA is 62.0%, while the corresponding values of the LSC, SSC, and IR regions are 62.6%, 67.2%, and 61.4%.

In order to further clarify the phylogenetic position of *E. brevicornu*, plastome of seven representative genus *Epimedium* species was obtained from NCBI to construct the plastome phylogeny, with *Caulophyllum robustum* as an outgroup. All the sequences were aligned using MAFFT v.7.313 (Katoh and Standley 2013) and maximum likelihood phylogenetic analyses were conducted using RAxML v.8.2.11 (Stamatakis 2014). The phylogenetic tree shows that *E. brevicornu* clustered together with *Epimedium acuminatum* and then formed one clade with *Epimedium dolichostemon* in the genus *Epimedium* (Figure 1).

**Figure 1.**
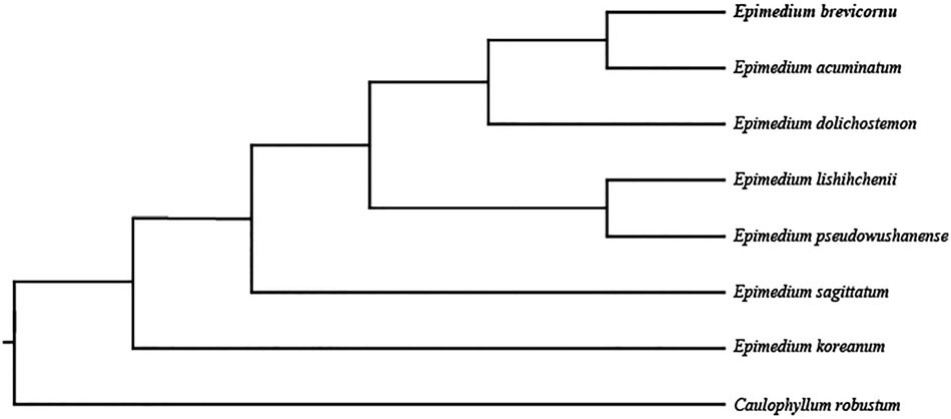
Phylogenetic relationships of Compositae species using whole chloroplast genome. GenBank accession numbers: *Epimedium acuminatum* (*NC_029941*); *Epimedium pseudowushanense* (*NC_029945*); *Epimedium lishihchenii* (*NC_029944*); *Epimedium koreanum* (*NC_029943*); *Epimedium dolichostemon* (*NC_029942*); *Epimedium acuminatum* (*NC_029941*); *Caulophyllum robustum* (*NC_042221*).

In summary, the complete cp genome from this study not only provides important insight into conservation and restoration efforts for *E. brevicornu*, but also plays a critical role in constructing a phylogeny of the genus *Epimedium*.
